# Attitudes, beliefs, and practices toward end-of-life care among physicians in sub-Saharan Africa

**DOI:** 10.11604/pamj.2023.45.167.40855

**Published:** 2023-08-18

**Authors:** Noah Rosenberg, Kondwelani John Mateyo, Kago Thuto Mokute, George Otieno, Kyle Hui, Elisabeth Riviello, Olivier Felix Umuhire

**Affiliations:** 1 University of Botswana, Department of Emergency Medicine, Gaborone, Botswana,; 2 University Teaching Hospital, Department of Internal Medicine, Lusaka, Zambia,; 3 African Inland Church Kijabe Hospital, Kijabe, Kenya,; 4 University of Hong Kong, Hong Kong, China,; 5 Harvard Medical School, Boston, Massachussets, United States of America,; 6 Beth Israel Deaconess Medical Center and Harvard Medical School, Boston, Massachussets, United States of America,; 7 Division of Clinical Services Northern Ontario School of Medicine University, Ontario, Canada

**Keywords:** Terminal care, life support care, withdrawing treatment, withholding treatment, Africa South of the Sahara, critical care, emergency treatment

## Abstract

**Introduction:**

as the opportunity to receive life-sustaining treatments expands in sub-Saharan Africa (SSA), so do potential ethical dilemmas. Little is known regarding the attitudes, beliefs, and practices of physicians in SSA regarding end-of-life care ethics.

**Methods:**

we used validated survey items addressing physician end-of-life care views and added SSA-context specific items. We identified a convenience sample using the authors' existing African professional contacts and snowball recruitment. Participants were invited via email to an anonymous online survey.

**Results:**

we contacted 78 physicians who practice critical care in Africa, and 68% (n=53) completed the survey. Of those, 66% were male, 55% were aged 36-45, 75% were Christian. They were from Kenya (30%), Zambia (28%), Rwanda (25%), Botswana (11%), and other countries (6%). Most (75%) agreed that competent patients can refuse even life-saving care. Only 32% agreed that their hospital had clear policies regarding withdrawing and withholding care, 11% agreed that their country had legal precedent for end-of-life care, and 43% believed that doctors could face legal or financial consequences for allowing patients to die by forgoing treatment. Pain control at the end of life, even if it may hasten death, was supported by 83%. However, 75% felt that clinicians undertreat pain due to fear of hastening death.

**Conclusion:**

participants strongly supported patient autonomy and end-of-life pain control but expressed concern that inadequate policy and legal frameworks exist to guide care and that pain is undertreated. Humane and actionable end-of-life care frameworks are needed to guide decisions in SSA.

## Introduction

An increase in life-sustaining technologies, including mechanical ventilation, hemodialysis, and artificial nutrition has made it possible for patients to remain alive for extended periods of time, while dependent on continuous interventions. These technologies became widely available in high-income countries (HIC) toward the end of the 20th century, forcing doctors, patients, and their family members to determine when to start life-sustaining measures, when to forgo them, and when to withdraw them. Health care professionals began to see that more intensive technological care was not always better care. By 1988 a recommendation to withhold or withdraw care preceded 51% of deaths in the United States (US) and that figure had risen to 90% by 1993 [1]. Even with this, doctors remained concerned that the therapies they provided were burdensome rather than beneficial to patients in some cases. In 1993, 47% of physicians surveyed in the US reported having acted against their conscience in providing aggressive care to the terminally ill [2]. Legal precedent, clear policy and consensus among ethicists progressed substantially in the following decades [3]. A patient's right to forgo or withdraw treatment, even if doing so would lead to death, was firmly established [4]. Ethicists arrived at near-consensus that there was no morally relevant distinction between withholding (not starting) and withdrawing (stopping once started) life-sustaining measures, though many physicians persisted in viewing the two as different [5]. The “doctrine of double effect,” the recognition that administration of medication with the intention of controlling pain at the end of life is acceptable, even if doing so hastens death, also gained widespread acceptance [5,6]. However, these developments largely occurred in HICs.

In low- and middle-income countries (LMICs), including sub-Saharan African (SSA) countries, resources for medical care are constrained, and availability of life-sustaining technology has developed more slowly. In 2020, Rwanda, Kenya, Botswana, and Zambia had 0.4, 1.0, 6.7, and 0.6 ICU beds/100,000 population, respectively [7], compared to 27 ICU beds/100,000 population in the US [8]. Critical care capacity is now rapidly expanding in SSA. In Zambia, the number of ICU beds/population more than doubled in the last decade [9]. With this welcome expansion of access to life-sustaining treatment in SSA, ethical dilemmas have emerged regarding its appropriate use and withdrawal for patients who are critically and terminally ill.

The attitudes and beliefs of doctors toward end-of-life care in the critically ill can differ substantially by region. Critical care physicians in Brazil, Japan, and Turkey were much less likely to recommend withdrawal of care for a patient in a vegetative state than their colleagues in Canada, Australia, and Northern Europe [10]. Some areas of international consensus may exist. In a 2014 survey of 1283 critical care physicians from 32 countries, there was greater than 80% agreement that, if a patient´s chances of surviving are extremely low, or the patient would not want continued life-sustaining treatment, therapy may be withheld or withdrawn [11]. However, this survey included only 11 responses from Africa, all of which were from South Africa. As capacity for critical care grows in SSA, it is essential that clear frameworks exist for end-of-life care in the critically ill that are appropriate for local cultural norms and beliefs. Yet little is known about physician attitudes, beliefs, and practices around end-of-life care in the critically ill in SSA. Our aim here was to describe the attitudes and beliefs of physicians practicing in SSA toward end-of-life care in the critically ill.

## Methods

### Survey development

The survey instrument was adapted from a previously validated survey of physicians in the US, including questions regarding patient autonomy, withdrawal, and withholding of care, and appropriateness of treatments at the end of life [2]. Questions regarding religion were added from the Duke University Religion Index, which is a validated scale that measures three domains, including organized religious activity (1-6 scale), non-organizational religious activity (1-6 scale) and subjective religiosity (1-15 scale), and can be used to compare relative religiosity between groups [12,13]. Additional questions on participant demographics, hospital characteristics, and legal and regulatory environment pertinent to the African context were developed de novo by the authors, based on their knowledge of SSA contexts. The draft survey was reviewed by 8 professionals with expertise in one or more areas relevant to the study, including survey methodology, end-of-life care, bioethics, and critical care in sub-Saharan Africa. These individuals were not involved in the development of the initial survey. Their feedback on face validity, completeness, ambiguity, format, and length of the survey were incorporated into subsequent versions in an iterative process. The final version of the survey was formatted for multiple choice and 5-point Likert scale responses on Qualtrics (see appendix).

### Participant recruitment and data collection

Participants included attending (consultant) or resident (registrar) physicians and surgeons who practice medicine in a critical care setting in sub-Saharan Africa. A critical care setting was defined as having a 1: 3 nurse-to-patient ratio or more, use of invasive or noninvasive positive pressure ventilation, acute hemodialysis, acute artificial nutrition, or continuous infusion of physiologically active medications (i.e. vasopressors, antihypertensives, insulin, etc.) Participants less than age 18 years, without proficiency in written English, or declining to participate were excluded. A convenience sampling approach was used for participant recruitment. A list of potentially qualifying doctors was generated by a study author familiar with critical care in each of the four SSA countries (OU for Rwanda, GO for Kenya, KJM for Zambia and KM for Botswana). The critical care resources and absolute number of physicians practicing in critical care settings in these countries is relatively low and an attempt was made to generate the most comprehensive list of eligible physicians possible.

Potential participants each received a personally addressed email inviting them to take part in the survey via an anonymous link in June 2021. Potential risks and benefits were described in the email. Participants were informed that taking the survey was voluntary and that they could stop at any time, and they consented by clicking on the survey link. Following completion of the survey, participants were asked to identify any colleagues who might also qualify for the survey. At the end of the survey, participants were directed to a separate site where they had the option to enter a drawing to win an Apple iPad. After one week, those who had not entered the iPad drawing were sent a follow-up reminder email. After 12 weeks, the survey was closed. Participant responses were downloaded from Qualtrics (Qualtrics, Provo, UT, USA) for statistical analysis. The project was approved by the Lifespan Inc. Institutional Review Board in Providence, Rhode Island, USA 207520 45CFR 46.101(2).

### Statistical methods

Participant and hospital characteristics were reported as exact proportions of categorical variables. Survey items representing participant attitudes, beliefs and practices were reported as proportions, with 95% confidence interval, of those indicating agree/strongly agree or those indicating frequently/always. All statistical analysis was conducted with Stata/IC 15.1 (StataCorp, College Station, TX).

## Results

### Participant characteristics

Invitations were initially sent to 68 physicians and an additional 10 were referred by participants. No response was received from 20 (25%) participants and 5 (8.6%) started but did not complete the survey, resulting in 53 (68%) of those invited completing all parts of the survey. All participants were doctors who practice in SSA and care for critically ill patients. Participants were predominantly male (66%) and aged 36-45 (55%). Practice locations included Rwanda (25%), Kenya (30%), Zambia (28%), Botswana (11%) and other African countries (6%). Participants identified their religious affiliation as Protestant (38%), Catholic (32%), other Christian (13%). Duration of medical practice was predominantly 6-10 years (28%). Seventy-five percent (75%) reported having had a friend or close family member die prematurely due to inadequate medical care. Seventy-five percent (75%) of participants had taken formal ethics classes and 92% frequently use principles of autonomy, non-maleficence, beneficence and justice (Table 1).

**Table 1:** participant demographics and background

Age		Sex	
26-35	18 (34%)	Male	35 (66%)
36-45	29 (55%)	Female	18 (34%)
46-65	6 (11%)		
**Geographic and linguistic background**			
	Country of Birth	Country of Medical School	Country of practice
Rwanda	13 (25%)	11 (21%)	13 (25%)
Kenya	11 (21%)	10 (19%)	16 (30%)
Zambia	13 (25%)	13 (25%)	15 (28%)
Botswana	6 (11%)	1 (2%)	6 (11%)
Other	10 (19%)*	18 (34%)†	3 (6%)‡
**Language spoken at home**			
Kinyarwanda	12 (23%)		
Swahili	4 (8%)		
French	2 (4%)		
English	25 (47%)		
Setswana	5 (9%)		
Not specified	5 (9%)		
**Personal medical background**		Somewhat or strongly agree	
Throughout my life I have generally had good access to advanced medical care.		57%	
I have had family members or close friends die prematurely due to inadequate medical care.		74%	
**Professional medical background**			
**Years of practice since medical school**		**Medical specialty**	
<2	2 (4%)	General practitioner	2 (4%)
2-5	7 (13%)	Surgery	3 (6%)
6-10	15 (28%)	Internal Medicine	12 (23%)
11-20	25 (47%)	Critical Care	16 (30%)
>20	4 (8%)	Emergency Medicine	19 (36%)
		Hospice or palliative care	1 (2%)
Religion			
Protestant Christian	20 (38%)		Duke University Religion Index
Catholic Christian	17 (32%)	Organizational religious activity (1-6 scale)	3.8
Other Christian	7 (13%)	Non-organizational religious activity (1-6 scale)	3.2
Muslim	3 (6%)	Intrinsic religiosity (1-15 scale)	11.9
None	4 (8%)		
Other	2 (4%)		
**Prior ethics training**		Somewhat or strongly agree	
I have taken formal classes in medical ethics.		75%	
I frequently refer to principles of autonomy, justice, beneficence and non-maleficence.		92%	

* Democratic Republic of Congo (n = 2), Uganda (n = 1), not specified (n = 7) † USA (n=5), Democratic Republic of Congo (n = 3), Europe (n = 1), Uganda (n = 1), not specified (n = 8) ‡ Uganda (n = 1), Democratic Republic of Congo (n = 1), not specified (n = 1)

### Hospital and practice setting characteristics

Participants practiced predominantly in hospitals with an ICU capacity of 6-10 beds (45%) and hospitals that required their patients’ family to cover the costs of treatments (34%). Mechanical ventilation and artificial nutritional support were always or frequently available for 83% of respondents. Some (21%) agreed that financial constraints limited patient care, with 57% of participants stating that cost is rarely considered when deciding what tests and treatments to order. Frequent supply shortages impacting care was reported by 26%. Participants agreed that withholding or withdrawing treatment from critically ill patients was mostly due to lack of benefit to the patient (66%), and also the possibility of treatment causing pain or suffering (43%). Care was sometimes withheld due to insufficient funds (19%), and unavailability of further care at that hospital (19%) (Table 2).

**Table 2:** participant's hospital of practice characteristics

Number of ICU beds in hospital of practice	
0-5	7 (13%)
6-10	24 (45%)
11-15	11 (21%)
16-20	7 (13%)
>20	4 (8%)
**Payer in hospital of practice**	
Patient's family	18 (34%)
Private insurance	10 (19%)
Government insurance	10 (19%)
No charge, government funded	15 (28%)
**Availability of life-support treatments**	
How frequently are the following treatment modalities available at your hospital?	Always or frequently
Mechanical ventilation	83%
Hemodialysis	57%
Infusion pumps	74%
Nutritional support	83%
	Agree or strongly agree
Supply shortages frequently impact patient care at my hospital	26%
Patient care is often limited at my hospital due to financial constraints	21%
I rarely consider cost when deciding what tests and treatments to order	57%
When treatments or interventions are withdrawn or withheld for a critically ill patient at your hospital, how often do you think the following is a primary reason?	Agree or strongly agree
Would have little or no benefit to the patient	66%
Would cause too much pain or suffering to the patient	43%
Inadequate funds for further care	19%
Further care not available at this hospital	19%

### Legal climate and hospital policy

Only 32% (CI: 20-46%) of participants agreed that their hospital had a clear and helpful policy regarding withdrawing and withholding care, and only 11% (CI: 4-23%) agreed that their country had clear legal precedent providing guidance in appropriate end-of-life care. Forty-three percent (43%), (CI: 30-58%) of participants believed that doctors could face financial penalty, loss of license, and/or criminal prosecution if they allowed patients to die by forgoing treatment; 53% (CI: 39-67%) believed that the same consequences could apply if doctors allowed patients to die by stopping treatment after it had been started; and 30% (CI: 18-44%) agreed that they knew of other physicians who had faced punishment for allowing patients to forgo or stop care.

### End-of-life decision-making and patient autonomy

When considering decisions regarding the use of life-sustaining treatments in critically ill patients, 70% (CI: 56-82%) of participants believed that the patient herself or himself was the most appropriate decision-maker. Forty-two percent (42%), (CI: 28-56%) were satisfied with how informed patients are regarding different care alternatives to life-sustaining treatments. Only 25% (CI: 14-38%) of the participants reported that resuscitation orders such as “do not resuscitate” or “full code” were routinely established as part of patient care, but 83% (CI: 70-92%) reported that meetings to discuss goals of care with the patient, doctors, and family members were routinely held in their practice.

### Physician attitudes and beliefs about appropriate treatments at the end of life

Participants reported some concern for inappropriate use of treatments at the end of life: 8% (CI: 2-18%) for mechanical ventilation, 26% (CI: 15-40%) for CPR, 19% (CI: 9-32%) for artificial nutrition and hydration. Nearly all (98%, CI: 90-100%) agreed that it is possible to prevent dying patients from feeling much pain. Eighty-three percent (83%), (70-92%) agreed that it is occasionally appropriate to give pain medication to relieve suffering, even if it may hasten the patient's death. However, 75% (62-86%) agreed that clinicians give inadequate pain medication out of fear of hastening a patient's death, and 79% (CI: 66-89%) of participants said that the most common form of narcotic misuse in the care of the dying was undertreatment of pain. Forty-nine percent (49%), (CI: 35-63%) of participants reported that they had acted against their conscience in providing care to the terminally ill, with 66% (CI: 52-78%) saying that the treatments they offer their patients are overly burdensome, and 42% (CI: 28-56%) that they gave up on patients too soon.

### Withholding and withdrawing treatment at the end of life

Seventy-five percent (75%), (CI: 62-86%) of participants agreed that competent patients have the right to refuse life support, even if that refusal may lead to death. Sixty percent (60%), (CI: 46-74%) agreed that dying patients should determine the best dosage regimen to control their pain, and 75% (CI: 62-86%) agreed that allowing patients to die by forgoing or stopping treatment is ethically different from assisting in suicide. Only 21% (CI: 11-34%) of participants agreed that there is no ethical difference between forgoing a life support measure and stopping it once it has been started, but 47% (CI: 33-61%) agreed that there is an emerging consensus among ethicists that withdrawing a treatment is not ethically different from withholding it (Table 3).

**Table 3:** physician attitudes and beliefs towards end-of-life care

Legal climate and hospital policy	Agree or strongly agree (CI)	Appropriate treatments at the end of life	Agree or strongly agree (CI)
My hospital has a clear and helpful policy regarding withdrawing and withholding care.	32% (20-46%)	For the critically and terminally ill patients you care for, how often are you concerned that the following treatments are used inappropriately:	
My country has clear legal precedent providing guidance in appropriate end-of-life care.	11% (4-23%)	Mechanical ventilation	8% (2-18%)
To allow patients to die by forgoing (not starting) treatment can result in the doctor being subject to financial penalty, loss of license and/or criminal prosecution.	43% (30-58%)	CPR	26% (15%-40%)
To allow patients to die by stopping treatment once started can result in the doctor being subject to financial penalty, loss of license and/or criminal prosecution.	53% (39-67%)	Artificial nutrition and hydration	19% (9-32%)
I know of other physicians who have faced punishment for allowing patients to forgo or stop care.	30% (18-44%)	Dialysis	6% (1-16%)
		Antibiotics	34% (22-48%)
		Pain medication	4% (0-13%)
**End-of-life decision making and patient autonomy**	Agree or strongly agree (CI)		
For decisions regarding the use of life-sustaining treatments in critically ill patients, how often do you think that the following person is the most appropriate decision maker? Assume the patient's mental status is uncompromised.		Sometimes it is appropriate to give pain medication to relieve suffering, even if it may hasten the patient's death.	83% (70-92%)
The patient himself or herself	70% (56-82%)	It is possible to prevent dying patients from feeling much pain.	98% (90-100%)
The treating doctor	40% (26-54%)	Clinicians give inadequate pain medication most often out of fear of hastening a patient's death.	75% (62-86%)
The head of the family	23% (12-36%)	The most common form of narcotic misuse in the care of the dying is undertreatment of pain.	79% (66-89%)
A community or religious leader	2% (0-10%)	At times, I have acted against my conscience in providing care to the terminally ill.	49% (35-63%)
		Sometimes I feel the treatments I offer my patients are overly burdensome.	66% (52-78%)
How satisfied are you with the extent to which . . .		Sometimes I feel we give up on patients too soon.	42% (28-56%)
Patients are informed of different care alternatives.	42% (28-56%)		
Patients understand the information they are told about their condition and treatment alternatives.	38% (25-52%)	**Withholding and withdrawing treatment at the end of life**	Agree or strongly agree (CI)
		All competent patients, even if they are not considered terminally ill, have the right to refuse life support even if that refusal may lead to death.	75% (62-86%)
Patients get the help they need to make decisions about care alternatives.	38% (25-52%)	To allow patients to die by forgoing or stopping treatment is ethically different from assisting in their suicide.	75% (62-86%)
Staff find out what critically and terminally ill patients want.	28% (17-42%)	There is no ethical difference between forgoing (not starting) a life support measure and stopping it once it has been started.	21% (11-34%)
Patients' wishes are recorded in the medical record.	26% (15-40%)	There is an emerging consensus among ethicists that withdrawing a treatment is ethically different from withholding or not starting it.	47% (33-61%)
Ethical issues in a patient's care are discussed by staff.	43% (30-58%)	The distinction between extraordinary (or “heroic”) measures and ordinary treatments is helpful in making termination-of-treatment decisions.	60% (46-74%)
		Disconnecting a feeding tube, even with consent from the patient and family, is killing a patient.	28% (17-42%)
Resuscitation orders such as “do not resuscitate” or “full code” are routinely established as part of patient care.	25% (14-38%)	Even if life support such as mechanical ventilation and dialysis are stopped, food and water should always be continued.	68% (54-80%)
Meetings to discuss goals of care with the patient, doctors and family members are routinely held.	83% (70-92%)	The burdens of continuing nutrition and hydration to a terminally ill patient can outweigh the benefits of prolonging life.	49% (35-63%)
		Dying patients should determine the best dosage regimen to control their pain.	60% (46-74%)

## Discussion

Our objective was to describe the attitudes and beliefs of physicians practicing in SSA toward end-of-life care in the critically ill. We found that among those polled there was widespread support for patient autonomy in decision making, including withdrawal and withholding of care, but also concern that a lack of regulatory and legal guidance surrounding end-of-life care in SSA puts physicians at personal risk.

End-of-life care is an inherent part of critical care medicine and difficult decisions regarding when to initiate and discontinue life-sustaining treatments for critically ill patients become more complex and common as medical advances proliferate and access expands. The resulting ethical dilemmas are prevalent in sub-Saharan Africa and are likely to increase. We describe the attitudes, beliefs, and practices of physicians practicing in sub-Saharan Africa toward the ethics of end-of-life care dilemmas. Our sample included physicians practicing medicine in multiple countries, men and women, a wide range of age groups, countries of origin, languages spoken, religions, training backgrounds, experience levels, and medical practice settings.

We found a concerning lack of regulatory and legal guidance for physicians. Only 32% of participants agreed that their hospital had a clear policy on withholding and withdrawing care, and only 11% agreed that their country had a legal precedent for these cases. However, 75% thought that competent patients have a right to refuse life support and that pain is undertreated for fear of hastening death. Doctors may find themselves in an impossible position: they believe that removing or withholding life-sustaining treatment for the critically ill, or aggressively treating pain, is the best care and desired by the patient, and yet they fear personal legal repercussions if they act in accordance with their convictions. Poor end-of-life care, inappropriate allocation of scarce critical care resources, and moral distress among physicians could result. Indeed, 66% of participants thought that the life-sustaining treatments they offered were sometimes overly burdensome.

Before legal precedent for the right to withdraw care was established in HICs, physicians did sometimes face legal risk. In 1975 the family of a patient with severe hypoxemic brain injury in the US requested that she be removed from the ventilator, but doctors declined the request when threatened with murder charges from the local prosecutor [14]. Only after the family successfully sued was mechanical ventilation discontinued. In 1983, two physicians, also in the US, were charged with murder after discontinuing mechanical ventilation and artificial hydration for a patient in a persistent vegetative state, at the request of family members and in accordance with his previously stated issues [15,16]. They were eventually acquitted.

These cases, and others like them, serve as crucial protections for physicians in HICs, but few equivalent court cases exist to provide precedent and protect physicians who withdraw care in SSA. At least one case in South Africa upheld the legality of withdrawal of care for a terminally ill patient [17], however experts in the country consider the question unresolved [18]. Elsewhere in SSA even less legal guidance exists. Hospitals, professional societies, and governments all need to participate in providing guidance and legal frameworks for end-of-life care.

In many respects, participants in this study expressed similar views to their colleagues elsewhere in the world. They were well-versed in common paradigms of medical ethics, with 75% reporting having taken ethics classes and 92% reported using the four principles approach [19]. Physicians expressed strong support for patient autonomy with 70% agreeing that the patient is the most appropriate decision maker. Most agreed with the doctrine of double effect (75%), allowing for medications to be offered for pain control even if the medications hasten death. Despite a growing consensus among ethicists that there is no ethically significant distinction between forgoing life-support and withdrawing it, our study revealed that 79% of physicians disagree with the ethicists. Similarly in the US, 59% of physicians viewed withdrawing life-sustaining treatment as more ethically problematic than forgoing treatment [20].

This study has some important limitations. Our sample size is small with only 53 participants; however, the number of physicians who regularly practice critical care medicine in Rwanda, Botswana, Zambia and Kenya is also very few compared to HIC countries, and we believe we were able to capture a large proportion of qualifying physicians by using the authors´ practice networks. We are unaware of any similarly-sized or larger studies of physician attitudes, beliefs, and practices regarding end-of-life care in SSA. In addition, our attention is limited to physicians, while nurses, patients, and family members are clearly vital stakeholders in the consensus building which must take place, and future work should include these groups. The particular survey methodology of this study is a strength, in that it included relevant background on participants´ demographics and experiences, as well as in-depth and validated questions on beliefs and practices.

## Conclusion

Ethical frameworks for addressing end-of-life care in SSA are needed. Physicians practicing in SSA recognize the need for end-of-life care that respects patient preferences and minimizes suffering, but also note the lack of institutional and legal frameworks to guide this care. Further work in eliciting other stakeholder views and developing guidelines is urgently needed.

### What is known about this topic

- *Life support technology can lead to ethical dilemmas in end-of-life care for the critically ill;*
- *Legal cases in high-income countries have contributed to precedent and guidance for physicians facing choices regarding withdrawal and withholding of care;*
- *Attitudes and beliefs of physicians toward ethical dilemmas in end-of-life care differ by region, but little is known regarding physicians practicing in sub-Saharan Africa*.

### What this study adds

- *Physicians practicing critical care medicine in sub-Saharan Africa strongly support patient autonomy and pain control at the end of life;*
- *They express concern for inadequate legal and regulatory guidance governing care at the end of life and fear for personal repercussions for withholding and withdrawing care, even when requested by patients and their family*.
